# Effects of Supplementation of Microalgae (*Aurantiochytrium* sp.) to Laying Hen Diets on Fatty Acid Content, Health Lipid Indices, Oxidative Stability, and Quality Attributes of Meat

**DOI:** 10.3390/foods9091271

**Published:** 2020-09-10

**Authors:** Bing Liu, Jiang Jiang, Dongyou Yu, Gang Lin, Youling L. Xiong

**Affiliations:** 1 State Key Laboratory of Food Science and Technology, Jiangnan University, Wuxi 214122, Jiangsu, China; bingliu08@zju.edu.cn (B.L.); Jiangjiang@jiangnan.edu.cn (J.J.); 2 College of Animal Sciences, Zhejiang University, Hangzhou 310058, Zhejiang, China; 3 Institute of Quality Standards and Testing Technology for Agricultural Products, Chinese Academy of Agricultural Sciences, Beijing 10081, China; lingang@caas.cn; 4 Department of Animal and Food Sciences, University of Kentucky, Lexington, KY 40546, USA

**Keywords:** *Aurantiochytrium* sp., health lipid indices, oxidative stability, meat quality, laying hens

## Abstract

The present study is conducted to investigate the effects of dietary docosahexaenoic acid (DHA)-rich microalgae (MA, *Aurantiochytrium* sp.) on health lipid indices, stability, and quality properties of meat from laying hens. A total of 450 healthy 50-wk-old Hy-Line Brown layers were randomly allotted to 5 groups (6 replicates of 15 birds each), which received diets supplemented with 0, 0.5, 1.0, 1.5, and 2.0% MA for 15 weeks. Fatty acid contents and quality properties of breast and thigh muscles from two randomly selected birds per replicate (*n* = 12) were measured. The oxidative stability of fresh, refrigerated, frozen, and cooked meat was also determined. Results indicated that supplemental MA produced dose-dependent enrichments of long-chain n-3 polyunsaturated fatty acids (n-3 LC-PUFA), predominantly DHA, in breast and thigh muscles, with more health-promoting n-6/n-3 ratios (1.87–5.27) and favorable lipid health indices (*p* < 0.05). MA supplementation did not affect tenderness (shear force) and color (L*, a*, and b* values) of hen meat nor muscle endogenous antioxidant enzymes and fresh meat oxidation (*p* > 0.05). However, the n-3 LC-PUFA deposition slightly increased lipid oxidation in cooked and stored (4 °C) meat (*p* < 0.05). In conclusion, MA supplementation improves the nutritional quality of hen meat in terms of lipid profile without compromising meat quality attributes. Appropriate antioxidants are required to mitigate oxidation when such DHA-enriched meat is subjected to cooking and storage.

## 1. Introduction

The increased public consciousness of the health benefits of long-chain n-3 polyunsaturated fatty acids (n-3 LC-PUFA), especially docosahexaenoic acid (DHA), has elevated consumer preference for the products fortified with these desirable nutrients [1,2]. DHA-enriched eggs are considered to be the most successful and efficient vehicles to incorporate DHA into the human diet [3]. Microalgae (MA), the primary producers of DHA, have been proven to be one of the most promising ingredients for DHA-enriched egg production due to their higher enrichment efficiency, production sustainability, and better sensory qualities than the commonly-used fish oil [4]. In addition to the incorporation of DHA into eggs, dietary MA to hens also produces dose-dependent incorporation of DHA into muscles [3]. With millions of laying hens going unused as food every year, increasing hen meat with a “healthy appeal” into the supply chain might be a promising way to meet increasing global needs. The use of DHA-enriched meat from hens after DHA-egg production could increase the value and desirability of hen meat or its processed products, given that consumers are already willing to pay more for n-3 LC-PUFA-enriched products [2]. Hen meat has been historically popular and highly desired in many Asian cultures in the preparation of aromatic and savory broths, owing to its specific flavor and aroma [5]. In France, boiled older chickens are regarded as delicacies [6]. Additionally, various methods of processing DHA-enriched hen meat (e.g., sausages) have been suggested to improve consumer acceptability.

However, published MA-based innovation studies have primarily focused on broiler meat and pork [7,8]. Little is known about the potential impacts of MA on hen meat. To establish MA-fed hen meat as a nutritional and palatable meat protein source, it is important to evaluate the lipid composition, muscle quality characteristics, and oxidative stability of meat from hens. Therefore, the present study aims to investigate the impacts of dietary DHA-rich MA (*Aurantiochytrium* sp.) on fatty acid (FA) profiles and quality attributes of meat from laying hens. In addition, the oxidative stability of the raw and cooked DHA-enriched meat, with or without storage, is determined.

## 2. Materials and Methods

### 2.1. Chemical Analyses of Microalgae (Aurantiochytrium sp.)

Microalgae (*Aurantiochytrium* sp., Alltech Inc., Nicholasville, KY, USA) powder was analyzed for moisture, crude protein, ether extract, and total ash content based on the procedure of the Association of Official Analytical Chemistry (AOAC) [9]. The FA contents were determined using gas chromatography (GC) by the method described in Section 2.4.1. The carotenoid-based antioxidant pigment profiles were analyzed by the method previously described [10]. The chemical compositions and FA content of microalgae and soybean oil are presented in Table 1.

### 2.2. Diets and Experiment Design

The present protocols were approved by the Institutional Animal Care and Use Committee of Zhejiang University (SYKX-2016-0380), following the principles and specific guidelines of the European Union legislation (2010/63/EU Directive). A total of 450 healthy Hy-Line Brown (50-wk-old) hens were arbitrarily allotted to 5 dietary treatments, with 6 replicates of 15 birds each. Treatments included a corn–soybean meal diet (control) and 4 treatment diets with DHA-rich MA (*Aurantiochytrium* sp., Alltech Inc., Nicholasville, KY, USA) supplementation at a rate of 0.5%, 1.0%, 1.5%, and 2.0% for 15 weeks. MA were supplemented at the expense of soybean meal and soybean oil. All experimental diets (in the form of mash) were formulated to be isocaloric and isonitrogenous to meet the National Research Council (NRC) recommendations for laying hens (Table 2). The diets were prepared weekly and kept at 4 °C in airtight containers to prevent rancidity and oxidation. Diets were mixed in a horizontal ribbon mixer for 30 min, with MA being incorporated into a small amount of ground corn before inclusion to ensure accurate dispersal throughout the feed. Birds were kept in an environmentally controlled henhouse (temperature: 23 ± 2 °C; relative humidity: 55% to 75%), with 3-tier battery cage systems under 16:8 h light:dark cycles. All birds had free access to feed and clean water.

### 2.3. Sampling and Processing

At the end of the feeding trial, 60 randomly selected birds (2 hens per replicate, i.e., totaling 12 hens per treatment, *n* = 12) were euthanized by electrically stunning them following cervical dislocation. After exsanguination for 5 min, the breast and thigh muscles were excised and weighed to calculate the muscle yield relative to their live body weight. Then, the left half of the breast (*pectoralis major*) and thigh (*biceps femoris*) muscle was immediately stored at 4 ± 2 °C for the analysis of meat quality at 24 h postmortem (pH, color, and drip loss) and 6 d postmortem (drip loss). For the analysis of the oxidative stability of such DHA-enriched meat, the right side of the fresh breast and thigh muscle was subjected to the following processes: cooked (baked in an oven at 180 °C for 20 min), refrigerated storage (kept in Ziploc polypropylene bags at 4 °C for 6 days), and frozen storage (kept in Ziploc polypropylene bags at −20 °C for 3 months). Parts of samples were snap-frozen in liquid nitrogen for antioxidant status assay.

### 2.4. Fatty Acid Analysis

#### 2.4.1. Fatty Acid Content

Fatty acids in meat were determined by GC, and the FA methyl esters (FAMEs) were prepared by the methods we previously described [5]. In brief, FAs were extracted from the homogenized samples with a chloroform and methanol mixture (2:1, *v*/*v*). Approximately 0.2 mL of the Folch extracts, with an internal standard (undecanoic acid, C11:0, Nu-Chek-Prep Inc., Elysian, MN, USA), were saponified with 2 mL of 0.5 M KOH in methanol (90 °C for 15 min), followed by methylation with 1.5 mL of BF3/methanol (90 °C for 2 min). The quantification of FAMEs was conducted by GC (Agilent 6890A series, USA) equipped with an FID and an SP-2560 fused silica capillary column (100 m × 0.25 mm, 0.20 µm). The detailed testing procedure was described in our previous study [5]. FAMEs were identified by using a standard 37 FAME mixture, and the FA contents (expressed as mg per 100 g fresh meat) were quantitatively calculated using the method of [12].

#### 2.4.2. Estimation of Health Lipid Indices

Fatty acids in meat are grouped in classes based on the double bonds as saturated, unsaturated, monounsaturated, and polyunsaturated fatty acids (SFAs, UFAs, MUFAs, and PUFAs). The health lipid indices were measured considering the ratios of n-6/n-3, MUFA/SFA, UFA/SFA, and PUFA/SFA, and thrombogenic indices (TI), atherogenic indices (AI), and hypocholesterolemic to hypercholesterolemic ratio (h/H ratio). The AI, TI, and h/H ratio were estimated according to the following equations [13]: TI = SFA/(0.5 × MUFA + 0.5 × n-6 PUFA + 3 × n-3 PUFA + n-3/n-6), AI = (4 × C14:0 + C16:0)/UFA, and h/H = (C18:1 + PUFA)/(C14:0 + C16:0).

### 2.5. Determination of Muscle Endogenous Antioxidant Enzyme Activity

The muscle samples were homogenized in chilled buffer (*w*/*v*, 1:9, 0.05 M Tris–HCl, 1 mM EDTA, pH 7.0), and then centrifuged at 3500 rpm for 20 min at 4 °C [14]. The total superoxide dismutase (T-SOD) and glutathione peroxidase (GPX) activity in the supernatant were determined by their respective kits (A001 and A005, Nanjing Jiancheng Bioengineering Institute, Nanjing, China). Data for T-SOD and GPX were expressed as U/mg protein.

### 2.6. Measurement of Antioxidant Potential

Meat fillets (2.5 g) were homogenized in 10 mL ethanol for the filtrates. DPPH (1,1-di-phenyl-2-picrylhydrazyl) activity was estimated using the method of [15]. In brief, sample solutions (1.5 mL) and 1.5 mL DPPH solution (0.1 mM) were fully mixed. After incubation for 30 min in the dark, the absorbance was recorded at 517 nm wavelength using a UV–vis spectrophotometer (Hitachi UV-3100, Tokyo, Japan). The percentage of DPPH radical scavenging activity = 100 − 100 × (Absorbance of sample/absorbance of control).

ABTS (2, 2-azinobis-3-ethylbenzothiazoline-6-sulfonic acid) radical scavenging activity was estimated using the method described by [16]. In brief, the diluted ABTS stock solution (3 mL with the initial absorbance of 0.70 ± 0.02 at 734 nm) and 20 μL sample solutions were mixed and shaken vigorously, and then the absorbance recorded at 734 nm after 30-min incubation (30 °C). ABTS radical scavenging activity was calculated as follows: ABTS radical scavenging activity (%) = 100 × (A_b_ − A_t_)/A_b_, where A_b_ is the absorbance of the control and A_t_ is the absorbance of the sample.

### 2.7. Assessment of Lipid Oxidation

Lipid oxidation of the meat samples was determined as 2-thiobarbituric acid-reactive substances (TBARS) by the method of [17]. A molar extinction coefficient of 152,000 M/cm was used to calculate the TBARS values, and the data were expressed as mg malondialdehyde (MDA)/kg fresh meat.

### 2.8. Meat Quality Attributes

At 24 h postmortem, the pH of the breast and thigh muscles was measured in triplicate with a pH meter (HI99161, HANNA Instrument, Italy), and meat color were estimated with a colorimeter (CR–410, Konica Minolta Sensing, Inc., Japan) on the freshly cut surface of each sample, based on the CIE L*a*b* system (L* for lightness, a* for redness, and b* for yellowness). Drip loss at 24 h and 6 d postmortem was determined using the method described previously [5]. For cooked meat quality tests, breast and thigh fillets were baked in a 176 °C oven to an internal temperature of 75 °C. Cooked fillets were cut into three small strips per sample (1 × 1 × 2.5 cm), parallel to the muscle fiber direction, to determine the shear force with a Warner-Bratzler shear device attached to a texture analyzer (TA-XT Plus, Stable Micro System Ltd., Surrey, UK). The detailed testing procedure was described previously [18].

### 2.9. Statistical Analysis

Data obtained from a total of 12 individual hens (6 replicates × 2 birds, *n* = 12) per dietary treatment were analyzed by SPSS 23.0 Statistical Software (SPSS Inc., Chicago, IL, USA), with each individual bird as the experimental unit. Normal distribution was tested using the Kolmogorov–Smirnov test. One-way ANOVA was used to determine the effects of MA supplementation on different parameters. Comparisons among means were made by the Tukey test. Orthogonal polynomial contrasts were conducted to estimate the linear and quadratic effects of MA supplementation on different parameters. Statistical significance was considered at *p* < 0.05.

## 3. Results

### 3.1. Fatty Acid Content

Supplemental DHA-enriched MA produced dose-dependent enrichments of eicosapentaenoic acid (EPA, C20:5 n-3), DHA (C22:6 n-3), and total n-3 LC-PUFA (EPA + DHA) in breast meat (Table 3). Conversely, linear or quadratic reductions of linoleic acid (LNA, C18:2n-6, *p* < 0.001), arachidonic acid (ARA, C20:4n-6, *p* < 0.001), and total n-6 PUFA contents (*p*_linear_ < 0.001; *p*_quadratic_ = 0.033) were observed in response to the increased MA supplementation. The DHA-enriched breast meat yielded a 4.40- to 13.06-fold increase in DHA content over the control. There were no notable differences (*p* = 0.804) on the total FAs in breast muscle among treatments.

Dietary graded levels of MA supplementation linearly decreased (*p* < 0.001) the total FAs in thigh meat (Table 4). The total n-3 LC-PUFA, especially for DHA, linearly and quadratically increased (*p* < 0.05) while the n-6 PUFA linearly and quadratically declined (*p*_linear_ < 0.001; *p*_quadratic_ < 0.001) in thigh muscle as the MA inclusion levels increased. Compared with control, the DHA-enriched thigh meat yielded a 6.90- to 18.72-fold increase in DHA content. The gradual decrease (*p*_linear_ < 0.001; *p*_quadratic_ < 0.001) in total n-6 PUFA content was observed from 0% to 2% of MA, attributed to the progressively declined trend of LNA (*p*_linear_ < 0.001; *p*_quadratic_ = 0.004) and ARA (*p*_linear_ < 0.001; *p*_quadratic_ < 0.001).

### 3.2. Health Lipid Indices

Progressive increases in PUFA/SFA, UFA/SFA, and h/H ratios, as well as gradual reductions in n-6/n-3 ratio and TI, were observed in the breast muscle with the increasing levels of MA (Table 5). A similar tendency in the health lipid indices of the thigh meat was observed except for the nonsignificant effect on UFA/SFA (*p* = 0.155) and h/H ratios (*p* = 0.054). No statistical differences were found in the MUFA/SFA ratio (*p* = 0.517) and AI (*p* = 0.322) in both breast and thigh meat.

### 3.3. Antioxidative Potential

Supplemental MA quadratically increased the DPPH (*p*_breast_ = 0.044; *p*_thigh_ = 0.032) and ABTS (*p*_breast_ < 0.001; *p*_thigh_ = 0.002) free-radical-scavenging activities in both breast and thigh muscle (Table 6). Dietary MA (up to 1.5%) elevated (*p* < 0.001) the ABTS-reducing activity of hen muscle relative to the control, whereas no differences (*p* > 0.05) were found between the control and 2.0% MA groups. No significant dietary treatment effects were observed on the DPPH (*p*_breast_ = 0.178; *p*_thigh_ = 0.221), T-SOD (*p*_breast_ = 0.185; *p*_thigh_ = 0.751), or GPX (*p*_breast_ = 0.358; *p*_thigh_ = 0.442) activities in the breast and thigh muscle from the MA-supplemented hens, as compared to the control.

### 3.4. Lipid Oxidation

The TBARS values in both breast and thigh muscle after refrigerated storage or cooking showed a linear (*p* < 0.05) increase corresponding to MA supplementation (Table 7). No differences were observed in TBARS values of fresh breast meat across the treatments (*p* = 0.544). However, dietary supplementation with increasing MA (up to 2.0% MA) linearly increased (*p* < 0.001) lipid oxidation in fresh thigh.

### 3.5. Meat Quality Attributes

The increased inclusion levels of MA in hen diets linearly (*p* < 0.001) resulted in higher breast muscle yields (Table 8). Although the drip loss at 24 h post-mortem showed a linear increase (*p*_breast_ = 0.049; *p*_thigh_ = 0.040) as the MA-included levels increased, no statistical differences (*p*_breast_ = 0.301; *p*_thigh_ = 0.290) were found among the dietary treatments. However, significantly (*p* < 0.001) increased drip loss of breast and thigh meat were observed in the 2.0% MA treatment group relative to the control after refrigerated storage for 6 days. No significant effects (*p* > 0.05) of MA supplementation were observed on the pH value or color characteristics of the breast and thigh meat.

## 4. Discussion

The experimental results demonstrated dose-dependent accumulations of n-3 LC-PUFA, predominantly DHA, in both breast and thigh muscle of hens receiving 0–2.0% MA. Consistent with our findings, broilers fed DHA-rich microalgae, such as *Aurantiochytrium* sp., *N. oceanica*, and *Schizochytrium limacinum,* showed increased DHA contents in the breast or thigh muscle compared to those with a control ration [7,19,20]. The species of microalgae used in the current study (*Aurantiochytrium* sp.) produces DHA predominantly (Table 1). However, despite the fact that little EPA was detected in MA-supplemented diets, dose-dependent EPA enrichments in hen meat were found relative to control. The result is in accordance with the findings reported in broilers [20], which is likely attributed to the retro-conversion of DHA to EPA [21]. Concomitant with the increases of total n-3 PUFA in breast and thigh, the total n-6 PUFA contents (especially LNA and ARA) linearly decreased with MA supplementation. Our results are in accordance with several recent reports on pigs and broilers with MA supplementation [22,23]. The reduction in n-6 PUFA contents probably resulted from the competition of substrates and biosynthesis enzymes between the n-3 and n-6 PUFAs [24].

Food products that include at least 40 or 80 mg EPA + DHA per 100 g can be marketed as being a “source of n-3 PUFA” and “high in n-3 PUFA”, respectively [25]. In our present study, the supplementation of 0.5% or more *Aurantiochytrium* sp. to hen diets was responsible for the n-3 LC-PUFA (EPA + DHA) concentrations rising to 44.4–137.2 mg per 100 g hen meat, meeting the required standard to be considered a “source of n-3 PUFA” or to qualify as being “high in n-3 PUFA”. In this way, consumption of 100 g meat from hens fed 2.0% MA would provide a mean intake of more than 125 mg of n-3 LC PUFA, supplying about 50% of the daily recommended intake (250 mg per day) for n-3 LC-PUFA [25]. In comparison, the control hen meat provided only 5.0% of the recommended daily intake. The data reported in broilers also produced similar estimates of enrichment [26].

The nutritional properties of meat are largely attributed to its fat and FA content, and balanced FA intakes are crucial to decrease the risk of atherosclerosis, cardiovascular, and other related diseases [1]. Hence, health lipid indices based on the functional impacts of FAs were used in this study for nutritional evaluation of the n-3 LC-PUFA enriched meat [27]. It is well documented that a dietary PUFA/SFA ratio of above 0.45, coupled with an n-6:n-3 ratio below 4.0, is desirable for the protection of the human cardiovascular system [28]. In the present study, the increase in DHA contents subsequently resulted in a linear and quadratic reduction in the n-6: n-3 ratio, and a reverse trend was found in PUFA/SFA ratio in breast and thigh muscle. As the MA levels increased up to 2.0%, the n-6:n-3 ratio declined from 12.77 to 1.87 in breast meat and from 12.28 to 2.28 in thigh meat. Our results indicate that the meat from hens fed MA of 1.0% or more complies with the recommendations (below 4.0), while those fed basal or diets containing less than 1.0% MA in the current study do not.

AI and TI are vital parameters indicating the potential for stimulating platelet aggregation, and the h/H ratio is associated with cholesterol metabolism [29]. From the perspectives of human health, healthy animal products can be characterized by low AI and TI and a high h/H ratio. Generally, the desired AI and TI recommended for human consumption are less than 0.5 and 1.0, respectively [29]. In this study, the breast meat showed AI and TI of 0.42–0.43 and 0.60–0.82, and the thigh meat showed AI and TI of 0.42–0.44 and 0.62–0.80 with MA supplementation, which were within the recommended ranges. The lowest AI and TI were observed in meat from hens fed 2.0% MA. The better health lipid indices, with lower n-6:n-3 PUFA ratio, as well as higher PUFA:SFA and h/H ratios caused by dietary MA inclusion, were in good agreement with the findings of an earlier study in broilers [27]. Hence, meat from MA-fed hens could be categorized as “beneficial to human health consumption”, the consumption of which might help reduce the risk of atherosclerosis, cardiovascular, and other related diseases.

Chicken meat, in particular with n-3 PUFA enrichment, is highly prone to oxidative processes [30]. It has been shown that n-3 PUFA enrichment by the inclusion of fish or flaxseed oils in poultry diets reduced the oxidative stability of chicken meat [26,31]. However, the present study demonstrated that MA supplementations at less than 2.0% of the diets did not increase the TBARS values in fresh breast and thigh muscle, which is in good agreement with previous findings with *Schizochytrium* sp. [7] and defatted *N. oceanica* [32] to broilers. The discrepancies from fish or flaxseed oil-supplemented meat may be accredited to the antioxidant properties of microalgae. In addition to DHA enrichment, the *Aurantiochytrium* sp. are rich in betacarotene (Table 1), which is simultaneously transferred into the muscle with the microalgal DHA incorporation. The increased antioxidant components enhance the oxidative stability of muscle via the nonenzyme antioxidant system reflected by the increased DPPH and ABTS radical-scavenging activities. Similar beneficial effects of vitamins in MA on antioxidant status were found in broilers [7]. On the contrary, a previous study indicated that broilers receiving MA increased the susceptibility to oxidation of meat [26]. This can likely be explained by the insufficient radical-scavenging activities of the intrinsic antioxidant components to mitigate the lipid oxidation induced by the increased n-3 PUFA enrichment.

The processes of cooking and storage usually aggravate lipid peroxidation in poultry meat [33]. In comparison with the fresh raw breast and thigh meat, regardless of MA supplemental levels, increased TBARS values were observed in refrigerated and heat-processed meat. After 6 d of refrigerated storage, meat from MA-supplemented hens displayed higher instability compared to those from nonsupplemented birds. Moreover, the dietary n-3 PUFA had a stronger effect on heated meat than on fresh or frozen meat, which is in accordance with the findings of Eder et al. [33]. The supplementation with 2.0% MA to hen diets resulted in increases by 47.3% and 44.4% of the TBARS content in cooked breast and thigh meat relative to the control. In addition, thigh meat showed more susceptibility to peroxidation than breast meat, which is probably associated with the absolute higher intramuscular fat and PUFA content (Table S1) in thigh than breast muscle [34]. Overall, meat enriched with n-3 PUFA with MA supplementation showed reduced oxidative stability during processing or refrigerated storage, indicating that the intrinsic antioxidant components are not sufficient in mitigating lipid oxidation. Additional antioxidant supplementation appears to be a notable strategy for diminishing lipid oxidation of meat [35]. Recent studies have demonstrated that dietary inclusion of antioxidants such as vitamin E and plant/herb extracts could inhibit lipid peroxidation in fresh, cooked, or frozen stored chicken meat [36,37,38]. As such, additional antioxidants can be included in diets to prevent oxidative deterioration of DHA-enriched meat, especially meat subjected to the processes of cooking or storage.

The present study also demonstrated higher breast muscle yield in MA-supplemented hens than those of the control hens, which is possibly associated with the increased muscle protein synthesis stimulated by MA [39]. Similar beneficial effects of MA on muscle yield were also found in broilers [7]. In this sense, the MA-induced raise in the edible meat yield may encourage the interest of abattoirs slaughtering hens for meat production purposes. The increased inclusion levels of MA in hen diets linearly decreased the shearing force of both breast and thigh meat. Although not statistically different, there was a numerically lower shear force in the meat of birds fed MA rather than the control. Generally, the shear force of MA-treated hen meat ranged from 32 to 37 N, which is still regarded as tender (less than 45 N) by consumers [40]. There were no significant effects on water holding capacity (WHC, determined by the drip loss) of the breast and thigh muscle at 24 h postmortem, following the MA treatment. However, the drip loss of hen meat at 6 d postmortem was significantly decreased in a linear or quadratic response. The significant reduction in WHC during refrigerated storage might be due to the oxidative degradation of membrane phospholipids, which damages the structure and function of membranes and increases the loss of sarcoplasmic fluid [41]. On the contrary, previous studies indicated that MA supplementation decreased the drip loss of meat in broilers [7] and pigs [23]. This phenomenon was explained by low levels of n-3 PUFA enrichment that enabled the muscular cells to build a flexible lipid bilayer membrane, and thus resulting in an increased WHC. These results indicate that the effects of MA on meat quality properties are highly dependent on the included levels, microalgae species, and their chemical composition.

## 5. Conclusions

In addition to enrichment in eggs, dietary MA (*Aurantiochytrium* sp.) offers an opportunity to enrich n-3 LC-PUFAs in hen meat in a dose-dependent manner, particularly the nutritionally beneficial DHA. The DHA enrichments permitted the meat to be labeled as a “source of n-3 PUFA” or “high in n-3 PUFA”, with a more health-promoting n-6/n-3 ratio and favorable lipid health indices. Dietary supplementations with up to 2.0% MA had a minimal impact on oxidative stability and meat quality attributes of fresh breast and thigh meat. However, the oxidative stability was slightly decreased when such “high in n-3 PUFA” meat was subjected to the processes of cooking and refrigerated storage. Future research should focus on evaluating MA in combination with additional antioxidants to mitigate oxidation during meat storage and processing.

## Figures and Tables

**Table 1:** Proximate chemical composition and fatty acid profile of microalgae (*Aurantiochytrium* sp.) and soybean oil used in the present study.

Items	MA (*Aurantiochytrium* sp.) ^1^	Soybean Oil ^2^
**Proximate analysis, %**		
Moisture	2.5	1.0
Crude protein	13.2	0
Ether extract	67.5	98.0
Ash	4.2	0.5
Metabolic energy, Mcal/kg	6.06	8.37
**Fatty acid content, mg/g**		
C16:0 Palmitic acid	356.50	102.68
C18:0 Stearic acid	12.08	48.54
C18:1n-9 Oleic acid	2.05	210.65
C18:2n-6 Linoleic acid	0.76	515.62
C18:3n-3 α-linolenic acid	10.52	78.54
C20:5n-3 Eicosapentaenoic acid	1.50	0.15
C22:6n-3 Docosahexaenoic acid	184.50	0.36
**Antioxidant pigments, μg/g**		
Total carotenoids	1505.4	/
β-carotene	984.6	/
Astaxanthin	421.5	/
Canthaxanthin	99.2	/

^1^ The metabolic energy (ME) of microalgae (*Aurantiochytrium* sp.) is 6.06 Mcal/kg [11]. ^2^ The proximate analysis and metabolizable energy of the soybean oil were obtained from a Chinese feed database that provides tables of feed composition and nutritive values (28th edition, 2017).

**Table 2:** Ingredients, nutrient levels, and fatty acid content of the experimental diets for laying hens (air-dry basis).

Item	Control	0.5% MA	1.0% MA	1.5% MA	2.0% MA
**Ingredients, %**					
Corn, 8.0% CP	60.00	60.00	60.00	60.00	60.00
Soybean meal, 45% CP	26.00	25.80	25.60	25.40	25.20
Soybean oil	2.00	1.70	1.40	1.10	0.80
Microalgae	0	0.50	1.00	1.50	2.00
Calcium carbonate	8.50	8.50	8.50	8.50	8.50
Dicalcium phosphate	1.20	1.20	1.20	1.20	1.20
Salts	0.30	0.30	0.30	0.30	0.30
DL-Methionine	0.20	0.20	0.20	0.20	0.20
Lysine-HCl	0.05	0.05	0.05	0.05	0.05
Premix ^1^	1.75	1.75	1.75	1.75	1.75
**Nutrient levels ^2^**					
ME, Mcal/kg	2.85	2.85	2.85	2.85	2.85
Crude protein, %	16.72 (16.73)	16.72 (16.75)	16.72 (16.72)	16.72 (16.74)	16.72 (16.73)
Ether extract, %	4.76(4.74)	4.75 (4.73)	4.75 (4.76)	4.75 (4.74)	4.75 (4.76)
Lysine, %	0.89	0.89	0.89	0.89	0.89
Cysteine + Methionine, %	0.75	0.75	0.75	0.75	0.75
Calcium, %	3.70 (3.70)	3.70 (3.71)	3.70 (3.69)	3.70 (3.70)	3.70 (3.68)
Available phosphorus, %	0.38	0.38	0.38	0.38	0.38
**Fatty acid content, mg/g diet ^3^**					
C14:0	0.05	0.08	0.10	0.11	0.10
C16:0	6.15	7.68	8.85	10.13	11.52
C18:0	1.45	1.35	1.34	1.22	1.28
C18:1 n-9	8.25	8.03	7.84	7.68	7.42
C18:2 n-6	14.80	14.01	13.84	13.68	13.49
C18:3 n-3	0.66	0.58	0.64	0.67	0.74
C20:5 n-3	0	0.02	0.03	0.05	0.03
C22:6 n-3	0.08	0.93	1.84	2.68	3.65
SFA	7.65	9.10	10.29	11.46	12.90
MUFA	8.25	8.03	7.84	7.68	7.42
PUFA	15.54	15.54	16.35	17.08	17.91
n-3 PUFA	0.74	1.53	2.51	3.40	4.42

^1^ The premix provided the following nutrients per kilogram of diet: VA, 6250 IU; VD3, 3125 IU; VE, 15 IU; VK, 2 mg; thiamine, 1 mg; riboflavin, 8.5 mg; calcium pantothenate, 50 mg; niacin, 32.5 mg; pyridoxine, 8 mg; folate, 5 mg; VB, 125 mg; choline, 500 mg; phytase, 600 FTU; Fe, 60 mg; Cu, 8 mg; Mn, 65 mg; Zn, 60 mg; Se, 0.3 mg; I, 1 mg. ^2^ The values in parentheses indicate the analyzed value. Others are calculated values. The ME values were estimated from a Chinese feed database that provides tables of feed composition and nutritive values. ^3^ The values are analyzed value by GC. SFA (sum of saturated fatty acids) = C14:0 + C16:0 + C18:0; MUFA (sum of monounsaturated fatty acids) = C18:1 n-9; PUFA (sum of polyunsaturated fatty acids) = C18:2 n-6 + C18:3 n-3 + C20:5 n-3 + C22:6 n-3; n-3 PUFA = C18:3 n-3 + C20:5 n-3 + C22:6 n-3.

**Table 3:** Fatty acid content (mg/100 g fresh meat) of breast muscle from laying hens with graded levels of *Aurantiochytrium* sp. supplementation.

Item	Microalgae Supplemental Levels, %					*p*-Value		
	0	0.5	1.0	1.5	2.0	MA	Linear	Quadratic
Total fatty acid	1359 ± 52	1306 ± 69	1320 ± 93	1315 ± 120	1316 ± 66	0.804	0.640	0.912
C14:0	5.7 ± 0.5	5.8 ± 0.4	5.7 ± 0.3	5.5 ± 0.3	5.7 ± 0.3	0.511	0.488	0.635
C16:0	353.5 ±19.7	328.5 ± 26.9	345.1 ± 21.6	336.2 ±27.9	338.6 ± 20.7	0.121	0.310	0.259
C16:1 n-7	45.4 ± 4.0	43.6 ± 4.6	42.8 ± 5.8	40.9 ± 5.9	40.4 ± 5.1	0.125	0.009	0.821
C18:0	134.1 ± 7.2	132.5 ± 11.2	136.5 ± 9.7	127.3 ± 15.2	126.5 ± 9.3	0.120	0.043	0.322
C18:1 n-9	414.0 ± 27.2	423.8 ± 24.5	425.7 ± 39.1	418.0 ± 45.6	413.1 ± 26.5	0.849	0.807	0.287
C18:2 n-6	**262.1 ± 11.4 ^a^**	**243.7 ± 15.5 ^a,b^**	**229.7 ± 21.2 ^b^**	**208.0 ± 24.8 ^c^**	**200.5 ± 12.1 ^c^**	<0.001	<0.001	0.468
C18:3 n-3	15.8 ± 0.8	16.0 ± 2.4	14.9 ± 1.5	15.2± 2.5	14.6 ± 2.0	0.437	0.098	0.996
C20:4 n-6	**96.2 ± 5.3 ^a^**	**71.2 ± 4.6 ^b^**	**69.9 ± 5.7 ^b^**	**67.2 ± 4.9 ^b,c^**	**62.1 ± 4.0 ^c^**	<0.001	<0.001	<0.001
C20:5 n-3	**3.3 ± 0.4 ^d^**	**4.5 ± 0.6 ^c^**	**5.3 ± 0.7 ^c^**	**6.4 ± 1.4 ^b^**	**8.2 ± 1.0 ^a^**	0.001	<0.001	0.104
C22:6 n-3	**9.0 ± 1.4 ^e^**	**39.6 ± 2.7 ^d^**	**68.6 ± 5.8 ^c^**	**98.3 ± 4.9 ^b^**	**117.6 ± 4.8 ^a^**	<0.001	<0.001	<0.001
Partial sums of fatty acid ^1^								
SFA	493.2 ± 20.4	466.8 ± 32.3	487.3 ± 29.5	469.0 ± 39.7	470.7 ± 27.0	0.131	0.129	0.603
MUFA	459.3 ± 28.0	467.4 ± 27.0	468.5 ± 42.7	458.9 ± 47.5	453.4 ± 28.4	0.829	0.540	0.336
PUFA	386.4± 12.5	375.0 ± 18.1	388.4 ± 26.7	395.0 ± 36.2	402.9 ± 17.4	0.069	0.235	0.216
n-6 PUFA	**358.3 ± 13.1 ^a^**	**314.9 ± 18.7 ^b^**	**299.7 ± 25.0 ^b,c^**	**275.2 ± 33.1 ^c,d^**	**262.6 ± 14.6 ^d^**	<0.001	<0.001	0.033
n-3 PUFA	**28.2 ± 1.6 ^e^**	**60.0 ± 3.6 ^d^**	**88.7 ± 5.3 ^c^**	**119.8 ± 6.2 ^b^**	**140.4 ± 4.6 ^a^**	<0.001	<0.001	<0.001
n-3 LC-PUFA	**12.3 ± 1.1 ^e^**	**44.3 ± 2.8 ^d^**	**73.9 ± 5.9 ^c^**	**104.7 ± 5.9 ^b^**	**125.1± 4.5 ^a^**	<0.001	<0.001	<0.001

Values are means ± standard deviations (*n* = 12). Bold-faced means in the same row with different letters differ siGgnificantly (*p* < 0.05). ^1^ SFA (sum of saturated fatty acids) = C14:0 + C16:0 + C18:0; MUFA (sum of monounsaturated fatty acids) = C16:1 n-7 + 18:1 n-9; PUFA (sum of polyunsaturated fatty acids) = C18:2 n-6 + C18:3 n-3 + C20:4 n-6 + C20:5 n-3 + C22:6 n-3; n-6 PUFA = C18:2 n-6 + C20:4 n-6; n-3 PUFA = C18:3 n-3 + C20:5 n-3 + C22:6 n-3; LC-n-3 PUFA (sum of long-chain n-3 polyunsaturated fatty acids) = C20:5 n-3 + C22:6 n-3.

**Table 4:** Fatty acid content (mg/100 g fresh meat) of thigh muscle of laying hens with graded levels of *Aurantiochytrium* sp. supplementation.

Item	Microalgae Supplemental Levels, %					*p*-Value		
	0	0.5	1.0	1.5	2.0	MA	Linear	Quadratic
Total fatty acids	**2081 ± 114 ^a^**	**1979 ± 69 ^a,b^**	**1933 ± 77 ^b^**	**1931 ± 59 ^b^**	**1926 ± 119 ^b^**	<0.001	<0.001	0.108
C14:0	10.0 ± 0.4	10.1 ± 0.4	10.3 ± 0.8	10.3 ± 1.4	10.5 ± 1.8	0.846	0.272	0.836
C16:0	**558.1 ± 44.8 ^a^**	**528.4 ± 24.5 ^a,b^**	**510.3 ± 34.8 ^b^**	**506.2 ± 34.2 ^b^**	**501.5 ± 49.3 ^b^**	0.004	0.014	0.129
C16:1 n-7	69.6 ± 5.4	69.9 ± 4.0	65.6 ± 4.2	66.0 ± 5.8	64.7 ± 7.0	0.058	0.007	0.800
C18:0	154.9 ± 14.5	147.2 ± 11.3	146.6 ± 12.8	147.2 ± 12.6	142.3 ± 6.1	0.146	0.064	0.586
C18:1 n-9	672.7 ± 74.0	688.6 ± 41.5	665.2 ± 44.1	667.7 ± 37.2	663.6 ± 62.9	0.791	0.430	0.809
C18:2 n-6	**462.9 ± 23.7 ^a^**	**382.8 ± 19.4 ^b^**	**371.2 ± 26.9 ^b,c^**	**354.6 ± 20.4 ^c,d^**	**342.3 ± 20.4 ^d^**	<0.001	<0.001	0.004
C18:3 n-3	32.8 ± 2.7	31.0 ± 2.7	30.5 ± 2.1	31.4 ± 3.3	30.2 ± 4.8	0.341	0.114	0.483
C20:4 n-6	**103.8 ± 6.1 ^a^**	**66.3 ± 5.7 ^b^**	**51.0 ± 3.2 ^c^**	**40.4 ± 3.2 ^d^**	**38.9 ± 2.2 ^d^**	<0.001	<0.001	<0.001
C20:5 n-3	**6.7 ± 0.7 ^c^**	**7.6 ± 0.8 ^b^**	**9.0 ± 0.9 ^a,b^**	**9.6 ± 0.7 ^a^**	**10.0 ± 0.8 ^a^**	<0.001	<0.001	0.018
C22:6 n-3	**6.8 ± 0.8 ^e^**	**46.9 ± 4.0 ^d^**	**74.5 ± 4.2 ^c^**	**101.8 ± 3.3 ^b^**	**127.3 ± 8.9 ^a^**	<0.001	<0.001	0.040
Partial sums of fatty acid ^1^								
SFA	723.1 ± 46.6	685.6 ± 69.0	667.1 ± 40.1	663.7 ± 33.7	654.2 ± 50.1	0.056	0.013	0.630
MUFA	742.3 ± 71.2	758.5 ± 42.4	730.8 ± 43.1	733.7 ± 36.4	728.2 ± 66.2	0.647	0.286	0.828
PUFA	**613.0 ± 21.9 ^a^**	**534.8 ± 19.5 ^b^**	**536.1 ± 26.9 ^b^**	**537.8 ± 19.7 ^b^**	**548.6 ± 20.1 ^b^**	<0.001	<0.001	<0.001
n-6 PUFA	**566.7 ± 20.7 ^a^**	**449.1 ± 19.8 ^b^**	**422.2 ± 26.8 ^c^**	**395.0 ± 21.7 ^d^**	**381.2 ± 21.3 ^d^**	<0.001	<0.001	<0.001
n-3 PUFA	**46.3 ± 3.9 ^e^**	**85.7 ± 3.9 ^d^**	**114.0 ± 4.2 ^c^**	**142.9 ± 5.2 ^b^**	**167.4 ± 6.9 ^a^**	<0.001	0.001	<0.001
LC-n-3 PUFA	**13.5 ± 1.1 ^e^**	**54.7 ± 4.2 ^d^**	**83.3 ± 4.2 ^c^**	**111.4 ± 3.3 ^b^**	**137.2 ± 9.0 ^a^**	<0.001	<0.001	<0.001

Values are means ± standard deviations (*n* = 12). Bold-faced means in the same row with different letters differ significantly (*p* < 0.05). ^1^ SFA (sum of saturated fatty acids) = C14:0 + C16:0 + C18:0; MUFA (sum of monounsaturated fatty acids) = C16:1 n-7 + 18:1 n-9; PUFA (sum of polyunsaturated fatty acids) = C18:2 n-6 + C18:3 n-3 + C20:4 n-6 + C20:5 n-3 + C22:6 n-3; n-6 PUFA = C18:2 n-6 + C20:4 n-6; n-3 PUFA = C18:3 n-3 + C20:5 n-3 + C22:6 n-3; LC-n-3 PUFA (sum of long-chain n-3 polyunsaturated fatty acids) = C20:5 n-3 + C22:6 n-3.

**Table 5:** Health lipid indices of fresh chicken meat from laying hens with graded levels of *Aurantiochytrium* sp. supplementation.

Item ^1^	Microalgae Supplemental Levels, %					*p*-Value		
	0	0.5	1.0	1.5	2.0	MA	Linear	Quadratic
**Breast muscle**								
n-6/n-3 PUFA ratio	**12.77 ± 1.01 ^a^**	**5.27 ± 0.50 ^b^**	**3.39 ± 0.31 ^c^**	**2.30 ± 0.24 ^d^**	**1.87 ± 0.09 ^d^**	<0.001	<0.001	<0.001
PUFA/SFA ratio	**0.78 ± 0.03 ^c^**	**0.80 ± 0.05 ^b,c^**	**0.79 ± 0.03 ^c^**	**0.83 ± 0.02 ^a,b^**	**0.85 ± 0.03 ^a^**	0.003	0.001	0.348
MUFA/SFA ratio	0.93 ± 0.05	1.00 ± 0.05	0.96 ± 0.05	0.98 ± 0.04	0.96 ± 0.04	0.490	0.318	0.126
UFA/SFA ratio	**1.72 ± 0.06 ^b^**	**1.80 ± 0.09 ^a^**	**1.76 ± 0.06 ^a,b^**	**1.80 ± 0.07 ^a^**	**1.79 ± 0.06 ^a^**	0.001	0.012	0.362
Atherogenic index (AI)	0.44 ± 0.02	0.42 ± 0.03	0.43 ± 0.02	0.42 ± 0.02	0.43 ± 0.02	0.080	0.156	0.165
Thrombogenic index (TI)	**1.00 ± 0.04 ^a^**	**0.82 ± 0.05 ^b^**	**0.75 ± 0.02 ^c^**	**0.65 ± 0.03 ^d^**	**0.60 ± 0.02 ^d^**	<0.001	<0.001	0.005
h/H ratio	**2.23 ± 0.11 ^b^**	**2.40 ± 0.17 ^a^**	**2.32 ± 0.11 ^a^**	**2.38 ± 0.13 ^a^**	**2.37 ± 0.09 ^a^**	0.012	0.025	0.130
**Thigh muscle**								
n-6/n-3 PUFA ratio	**12.28 ± 0.72 ^a^**	**5.25 ± 0.37 ^b^**	**3.71 ± 0.28 ^c^**	**2.77 ± 0.22 ^d^**	**2.28 ± 0.18 ^e^**	<0.001	<0.001	<0.001
PUFA/SFA ratio	**1.03 ± 0.06 ^b^**	**1.11 ± 0.04 ^a^**	**1.10 ± 0.04 ^a^**	**1.11 ± 0.05 ^a^**	**1.11 ± 0.05 ^a^**	0.044	0.821	0.008
MUFA/SFA ratio	0.85 ± 0.05	0.78 ± 0.04	0.81 ± 0.08	0.81 ± 0.06	0.84 ± 0.07	0.517	0.841	0.494
UFA/SFA ratio	1.88 ± 0.07	1.89 ± 0.04	1.90 ± 0.10	1.92 ± 0.10	1.96 ± 0.07	0.155	0.071	0.573
Atherogenic index (AI)	0.44 ± 0.02	0.44 ± 0.01	0.44 ± 0.02	0.43 ± 0.03	0.42 ± 0.02	0.322	0.655	0.068
Thrombogenic index (TI)	**0.91 ± 0.04 ^a^**	**0.80 ± 0.03 ^b^**	**0.73 ± 0.04 ^c^**	**0.67 ± 0.03 ^d^**	**0.62 ± 0.03 ^e^**	<0.001	<0.001	0.001
h/H ratio	2.27 ± 0.12	2.27 ± 0.05	2.31 ± 0.12	2.34 ± 0.16	2.38 ± 0.13	0.054	0.013	0.687

Values are means ± standard deviations (*n* = 12). Bold-faced means in the same row with different letters differ significantly (*p* < 0.05). ^1^ n-6/n-3 PUFA ratio: n-6/n-3 polyunsaturated fatty acids ratio; PUFA/SFA ratio: polyunsaturated fatty acids/saturated fatty acids ratio; MUFA/SFA ratio: monounsaturated fatty acids/saturated fatty acids ratio; UFA/SFA: unsaturated fatty acids/saturated fatty acids ratio; h/H ratio: hypocholesterolemic/hypercholesterolemic ratio.

**Table 6:** DPPH and ABST free-radical-scavenging activity and antioxidant enzyme activity of fresh breast and thigh muscles in laying hens with graded levels of *Aurantiochytrium* sp. supplementation.

Item	Microalgae Supplemental Levels, %					*p*-Value		
	0	0.5	1.0	1.5	2.0	MA	Linear	Quadratic
**Breast muscle**								
DPPH, % of inhibition	20.18 ± 4.99	25.95 ± 6.10	23.75 ± 3.82	24.75 ± 7.84	23.88 ± 6.00	0.178	0.439	0.044
ABTS, % of inhibition	**42.14 ± 3.97 ^c^**	**48.10 ± 5.73 ^a^**	**51.31 ± 3.78 ^a^**	**47.38 ± 3.03 ^a,b^**	**42.62 ± 3.70 ^b,c^**	<0.001	0.950	<0.001
T-SOD, U/mg protein	70.34 ± 8.82	73.66 ± 6.10	75.33 ± 8.14	72.57 ± 6.22	68.47 ± 7.30	0.185	0.478	0.128
GPX, U/mg protein	3.35 ± 0.43	3.63 ± 0.46	3.54 ± 0.27	3.47 ± 0.41	3.32 ± 0.54	0.358	0.553	0.072
**Thigh muscle**								
DPPH, % of inhibition	16.23 ± 3.57	20.97 ± 4.19	21.90 ± 4.92	22.64 ± 4.88	20.29 ± 6.13	0.221	0.459	0.032
ABTS, % of inhibition	**39.40 ± 6.63 ^b^**	**45.83 ± 8.92 ^a^**	**45.71 ± 4.99 ^a^**	**44.76 ± 6.25 ^a^**	**42.86 ± 5.48 ^a,b^**	0.007	0.077	0.002
T-SOD, U/mg protein	85.90 ± 9.28	86.46 ± 6.19	86.22 ± 10.30	82.72 ± 5.45	85.38 ± 7.50	0.751	0.305	0.380
GPX, U/mg protein	3.51 ± 0.39	3.78 ± 0.52	3.66 ± 0.39	3.49 ± 0.40	3.62 ± 0.37	0.442	0.847	0.477

Values are means ± standard deviations (*n* = 12). Bold-faced means in the same row with different letters differ significantly (*p* < 0.05). DPPH: DPPH radical scavenging activity; ABTS: ABTS+ reducing activity; T-SOD: total superoxide dismutase; GPX: glutathione peroxidase.

**Table 7:** The biomarkers (TBARS, mg MDA/kg fresh meat) of lipid oxidation of fresh, refrigerated, frozen, and cooked meat from laying hens with graded levels of *Aurantiochytrium* sp. supplementation.

Item	Microalgae Supplemental Levels, %					*p*-Value		
	0	0.5	1.0	1.5	2.0	MA	Linear	Quadratic
**Breast muscle**								
Fresh	0.142 ± 0.023	0.150 ± 0.029	0.145 ± 0.027	0.155 ± 0.026	0.159 ± 0.031	0.544	0.130	0.832
Refrigerated storage	**0.272 ± 0.031 ^b^**	**0.294 ± 0.029 ^a,b^**	**0.304 ± 0.028 ^a,b^**	**0.315 ± 0.028 ^a^**	**0.309 ± 0.030 ^a^**	0.008	0.001	0.098
Frozen storage	0.166 ± 0.020	0.172 ± 0.015	0.163 ± 0.016	0.175 ± 0.018	0.186 ± 0.021	0.164	0.052	0.236
Cooked	**0.346 ± 0.017 ^c^**	**0.356 ± 0.022 ^c^**	**0.370 ± 0.018 ^b,c^**	**0.397 ± 0.032 ^b^**	**0.510 ± 0.022 ^a^**	<0.001	<0.001	0.596
**Thigh muscle**								
Fresh	**0.209 ± 0.031 ^b^**	**0.216 ± 0.013 ^a,b^**	**0.227 ± 0.019 ^a,b^**	**0.234 ± 0.019 ^a,b^**	**0.241 ± 0.025 ^a^**	0.006	<0.001	0.831
Refrigerated storage	**0.342 ± 0.016 ^c^**	**0.357 ± 0.018 ^b,c^**	**0.375 ± 0.022 ^a,b^**	**0.371 ± 0.015 ^a,b^**	**0.387 ± 0.022 ^a^**	<0.001	<0.001	0.355
Frozen storage	0.206 ± 0.024	0.217 ± 0.015	0.211 ± 0.018	0.220 ± 0.018	0.231 ± 0.020	0.242	0.045	0.584
Cooked	**0.423 ± 0.021 ^c^**	**0.443 ± 0.028 ^b,c^**	**0.450 ± 0.014 ^b^**	**0.476 ± 0.032 ^b^**	**0.611 ± 0.011 ^a^**	<0.001	<0.001	0.159

Values are means ± standard deviations (*n* = 12). Bold-faced means in the same row with different letters differ significantly (*p* < 0.05). TBARS, 2-thiobarbituric acid-reactive substances; MDA, malondialdehyde.

**Table 8:** Physical properties of fresh breast and thigh meat of laying hens (65-weeks old) with graded levels of *Aurantiochytrium* sp. supplementation.

Item ^1^	Microalgae Supplemental Levels, %					*p*-Value		
	0	0.5	1.0	1.5	2.0	MA	Linear	Quadratic
**Breast muscle**								
Breast muscle yield, %	**15.66 ± 0.83 ^b^**	**16.25 ± 0.84 ^a,b^**	**16.16 ± 0.75 ^a,b^**	**16.63 ± 0.89 ^a^**	**16.88 ± 0.63 ^a^**	0.005	<0.001	0.901
pH24 h	5.82 ± 0.15	5.78 ± 0.11	5.77 ± 0.15	5.80 ± 0.10	5.74 ± 0.16	0.699	0.306	0.946
Shear force, N	36.32 ± 3.63	35.57± 2.31	34.49± 2.51	34.38 ± 2.42	33.19 ± 2.71	0.270	0.034	0.487
L* value	50.29 ± 1.28	49.83 ± 1.19	49.18 ± 1.27	48.88 ± 1.32	49.22 ± 1.46	0.072	0.052	0.166
a* value	6.41 ± 1.08	6.05 ± 0.87	6.15 ± 0.63	6.79 ± 1.24	6.27 ± 0.93	0.399	0.611	0.845
b* value	12.42 ± 1.14	11.98 ± 1.38	13.02 ± 1.10	12.28 ± 0.94	13.18 ± 1.55	0.102	0.112	0.503
Drip loss, %								
After 1 days	2.80 ± 0.75	2.65 ± 0.45	2.90± 0.40	2.91± 0.36	3.11 ± 0.57	0.301	0.049	0.413
After 6 days	**8.08 ± 0.94 ^c^**	**8.64 ± 0.85 ^b,c^**	**9.21 ± 0.62 ^a,b^**	**9.24 ± 0.70 ^a,b^**	**9.93 ± 0.81 ^a^**	<0.001	<0.001	0.744
**Thigh muscle**								
pH24 h	6.09 ± 0.07	6.15 ± 0.14	6.09 ± 0.16	6.14 ± 0.11	6.07 ± 0.13	0.576	0.720	0.334
Shear force, N	35.07 ± 3.88	33.83 ± 2.95	31.89 ± 2.84	32.08 ± 2.58	32.27 ± 2.69	0.053	0.010	0.132
L* value	40.99 ± 2.90	39.66 ± 4.04	40.78 ± 3.58	38.58 ± 0.320	39.71 ± 1.74	0.357	0.216	0.644
a* value	16.44 ± 2.82	16.07 ± 1.82	15.72 ± 2.24	16.63 ± 1.73	15.68 ± 1.41	0.732	0.617	0.963
b* value	13.33 ± 2.08	13.14 ± 1.02	12.95 ± 1.15	13.30 ± 1.16	13.16 ± 1.27	0.965	0.886	0.676
Drip loss, %								
After 1 days	1.79 ± 0.36	1.87± 0.45	1.95 ± 0.24	2.06 ± 0.40	2.10 ± 0.37	0.290	0.040	0.674
After 6 days	**6.77 ± 0.55 ^b^**	**6.53 ± 0.33 ^b^**	**6.75 ± 0.45 ^b^**	**6.98 ± 0.38 ^b^**	**8.14 ± 0.49 ^a^**	<0.001	<0.001	<0.001

Values are means ± standard deviations (*n* = 12). Bold-faced means in the same row with different letters differ significantly (*p* < 0.05). L^*^ value, lightness; a^*^, redness; b^*^ value, yellowness.

## Supplementary Materials

The following are available online at https://www.mdpi.com/2304-8158/9/9/1271/s1. Table S1: Chemical composition (g/100 g) of breast and thigh meat from laying hens with graded levels of microalgae supplementation.
