# Supplementary material for: Effects of Supplementation of Microalgae (Aurantiochytrium sp.) to Laying Hen Diets on Fatty Acid Content, Health Lipid Indices, Oxidative Stability, and Quality Attributes of Meat
Source: Foods. 2020 Sep 10;9(9):1271. doi: 10.3390/foods9091271 (PMC7555786; doi:10.3390/foods9091271)
Supplement: Supplementary file 1 [file foods-09-01271-s001.pdf]

**Table S1.** Chemical composition (g /100 g) of breast and thigh meat from laying hens with graded levels of *Aurantiochytrium* sp. supplementation.

| Item | MA supplemental levels, % | | | | | *p*-value | | |
|---|---|---|---|---|---|---|---|---|
| | 0 | 0.5 | 1.0 | 1.5 | 2.0 | MA | Linear | Quadratic |
| **Breast muscle** | | | | | | | | |
| Moisture, % | 73.80 ± 0.59 | 73.68 ± 0.48 | 73.71 ± 0.42 | 73.74 ± 0.61 | 73.37 ± 0.70 | 0.408 | 0.133 | 0.422 |
| Crude protein, % | 22.74 ± 0.81 | 23.09 ± 0.59 | 23.12 ± 0.44 | 23.25 ± 0.66 | 23.47 ± 0.74 | 0.110 | 0.009 | 0.833 |
| Crude fat, % | **2.01 ± 0.17**[a] | **1.86 ± 0.23**[ab] | **1.74 ± 0.29**[ab] | **1.64 ± 0.27**[b] | **1.71 ± 0.20**[b] | 0.002 | < 0.001 | 0.079 |
| Ash, % | 1.45 ± 0.56 | 1.36 ± 0.27 | 1.43 ± 0.31 | 1.38 ± 0.35 | 1.33 ± 0.48 | 0.976 | 0.992 | 0.667 |
| **Thigh muscle** | | | | | | | | |
| Moisture, % | 75.00 ± 0.47 | 75.08 ± 0.48 | 75.06 ± 0.34 | 75.12 ± 0.48 | 75.15 ± 0.39 | 0.984 | 0.579 | 0.993 |
| Crude protein, % | **20.11 ± 0.66**[b] | **20.49 ± 0.45**[ab] | **20.65 ± 0.19**[ab] | **20.92 ± 0.34**[a] | **21.02 ± 0.39**[a] | 0.010 | < 0.001 | 0.524 |
| Crude fat, % | **3.76 ± 0.31**[a] | **3.35 ± 0.17**[b] | **3.19 ± 0.21**[bc] | **2.91 ± 0.14**[cd] | **2.80 ± 0.07**[d] | < 0.001 | < 0.001 | 0.129 |
| Ash, % | 1.12 ±0.19 | 1.08 ± 0.10 | 1.10 ± 0.33 | 1.06 ± 0.23 | 1.03 ± 0.22 | 0.986 | 0.606 | 0.918 |

Values are means ± standard deviations (n = 12). Bold-faced means in the same row with different letters differ significantly ($p < 0.05$).
